# Androgendeprivation als Initial- und Basistherapie beim Prostatakarzinom: Eine retrospektive Datenanalyse aus urologischen Praxen in Deutschland

**DOI:** 10.1007/s00120-024-02434-z

**Published:** 2024-08-29

**Authors:** Peter J. Goebell, Felix Cornelius, Annika Fernandez Milano, Sybill Hessler, Matthias Schulze

**Affiliations:** 1 Urologische und Kinderurologische Universitätsklinik, Friedrich-Alexander-Universität Erlangen-Nürnberg, Erlangen, Deutschland; 2 TumorScout GmbH, Berlin, Deutschland; 3 IQVIA Commercial GmbH & Co. OHG, Frankfurt am Main, Deutschland; 4 Ipsen Germany, München, Deutschland; 5 Praxis Dr. Schulze, Rathausstraße 33–35, 04416 Markkleeberg, Deutschland

**Keywords:** Androgendeprivationstherapie, Kastrationsresistentes Prostatakarzinom, Retrospektive Datenanalyse, Real-world-Daten, Versorgungsforschung, Androgen deprivation therapy, Castration-resistant prostate cancer, Retrospective data analysis, Real-world data, Health services research

## Abstract

**Hintergrund:**

Ziel dieser Studie war die Bestimmung des Anteils der Patienten mit einem Prostatakarzinom (PCa), die nach Beginn einer Therapie für ein kastrationsresistentes Prostatakarzinom (KRPCa) die primäre Androgendeprivationstherapie (ADT) beibehielten sowie die Beschreibung ihrer Behandlungsmuster.

**Methodik:**

Retrospektive Analyse von 609.308 Patienten in urologischen Praxen in Deutschland von 2011 bis 2020 auf Basis von anonymisierten Sekundärdaten des Webservers UROscience. PCa-Patienten waren für die Studie geeignet, wenn sie nach einer 6‑monatigen verschreibungsfreien Prä-Indexperiode eine ADT erhielten.

**Ergebnisse:**

Insgesamt wurden 3.112 Patienten (Durchschnittsalter: 75,5 [± 8,0] Jahre) eingeschlossen. Die meisten Patienten erhielten Gonadotropin-Releasing-Hormon (GnRH)-Agonisten (72,3 %), gefolgt von Antiandrogenen (24,9 %). Die mediane Dauer der ADT-Behandlung betrug 25,9 Monate. Die geschätzten Wahrscheinlichkeiten, die ADT 3, 6 und 8 Jahre nach Behandlungsbeginn fortzusetzen, lagen bei 40,7 %, 20,1 % bzw. 12,7 %. Eine Unterbrechung über alle ADT hinweg erfolgte bei 42,7 % der Patienten, eine Umstellung der primären ADT bei 52,2 % und ein Abbruch bei 82,2 % der Patienten. Nach Beginn der ADT erhielten 14,6 % der Patienten eine Therapie für KRPCa, von denen 76,4 % die primäre ADT fortsetzten. Die mediane Dauer der KRPCa-Behandlung betrug 11,0 Monate. Die geschätzten Wahrscheinlichkeiten, 3, 6 und 8 Jahre nach Beginn der ADT ein KRPCa zu entwickeln, lagen bei 11,1 %, 20,1 % und 25,9 %.

**Schlussfolgerung:**

Diese Studie hat gezeigt, dass bei einem relevanten Anteil der Patienten die primäre ADT nach Beginn der Therapie für KRPCa abgesetzt wurde, obwohl Leitlinien die Fortsetzung der ADT bei Fortschreiten der Erkrankung empfehlen.

**Graphic abstract:**

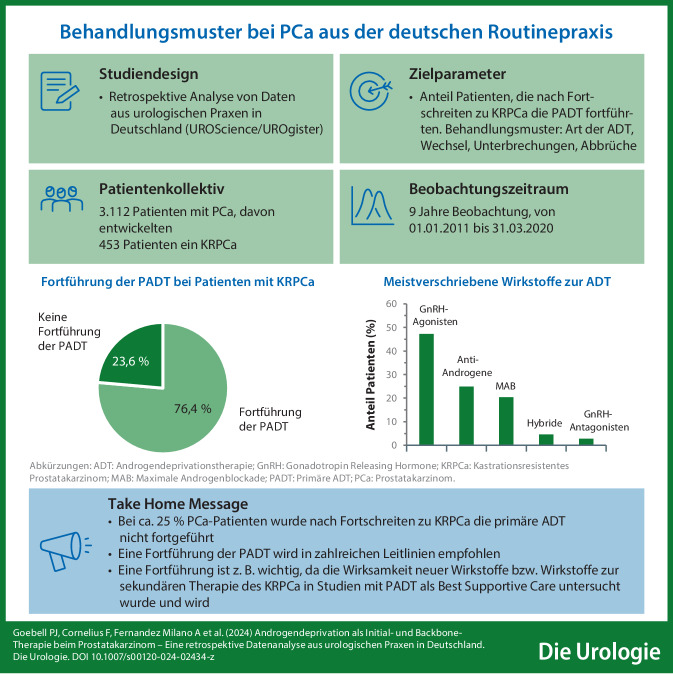

**Zusatzmaterial online:**

Die Online-Version dieses Beitrags (10.1007/s00120-024-02434-z) enthält weitere Abbildungen und Tabellen

## Abstract (en)

**Background:**

The aim of this study was to determine the proportion of patients with prostate cancer (PCa) who remained on primary androgen deprivation therapy (ADT) after starting treatment for castration-resistant prostate cancer (CRPC) and to describe their treatment patterns.

**Materials and methods:**

The study comprises a retrospective analysis of 609,308 patients in urological practices in Germany from 2011 to 2020 based on anonymized secondary data from the UROscience webserver. PCa patients were eligible for inclusion if they received ADT after a 6-month prescription-free pre-index period.

**Results:**

A total of 3,112 patients (mean age 75.5 [±8.0] years) were included. Most patients received gonadotropin-releasing hormone (GnRH) agonists (72.3%), followed by antiandrogens (24.9%). The median duration of ADT treatment was 25.9 months. The estimated probabilities of continuing ADT 3, 6, and 8 years after starting treatment were 40.7%, 20.1%, and 12.7%, respectively. Interruption across all ADTs occurred in 42.7% of patients, switching of primary ADT in 52.2% and discontinuation in 82.2% of patients. After starting ADT, 14.6% of patients received treatment for CRPC, of whom 76.4% continued primary ADT. The median duration of CRPC treatment was 11.0 months. The estimated probabilities of developing CRPC 3, 6, and 8 years after starting ADT were 11.1%, 20.1%, and 25.9%, respectively.

**Conclusion:**

This study has shown that a relevant proportion of patients discontinued primary ADT after starting treatment for CRPC, although guidelines recommend continuing ADT if the disease progresses.

Das Fortsetzen der primären Androgendeprivationstherapie (ADT) nach Einleiten einer sekundären Therapie des kastrationsresistenten Prostatakarzinoms (KRPCa) wird von mehreren internationalen Leitlinien empfohlen. Eine Querschnittanalyse von Daten aus europäischen Ländern hat jedoch gezeigt, dass diese Empfehlung in der Routinepraxis häufig nicht umgesetzt wird. Die hier präsentierte retrospektive Studie basierend auf Daten von 3.112 Patienten aus urologischen Praxen aus den Jahren 2011–2020 hat die Situation in Deutschland analysiert.

## Einleitung

Das Prostatakarzinom (PCa) ist der häufigste Urogenitaltumor bei Männern mit weltweit mehr als 1,4 Mio. Fällen und mehr als 375.000 damit verbundenen Todesfällen im Jahr 2020 [9, 17]. In Deutschland lag die Zahl der PCa-Neuerkrankungen im Jahr 2018 bei etwa 62.500 [8].

Zu den aktuellen Therapieoptionen gehören – je nach Krankheitsstadium – aktive Überwachung/Watchful Waiting, radikale Prostatektomie, Strahlentherapie, Hormontherapie, Chemotherapie, Immuntherapie und Radionuklidtherapie [13, 15, 18]. Die Hormontherapie bezieht sich in erster Linie auf die medikamentöse ADT, die eine Behandlung mit Gonadotropin-Releasing-Hormon (GnRH)-Agonisten, GnRH-Antagonisten oder Antiandrogenen (AA) umfasst [13]. Die ADT wird als Initial- oder Basistherapie bei nicht metastasierten und metastasierten hormonsensitiven (HSPCa) und kastrationsresistenten PCa (KRPCa) eingesetzt. Die meisten Patienten sprechen anfangs gut auf die ADT an, viele von ihnen entwickeln jedoch nach einigen Jahren eine Kastrationsresistenz [6].

Das KRPCa ist eine fortgeschrittene Form des PCa, die durch eine Progression der Erkrankung nach chirurgischer Kastration oder ADT gekennzeichnet ist. Die Prognose ist schlecht, und die Überlebenszeit ist im Vergleich zu kastrationsempfindlichen Patienten verkürzt [7]. Die geschätzte Prävalenz des KRPCa bei PCa-Patienten liegt bei etwa 18 % [7]. Von diesen Patienten haben 70 % Metastasen [12]. Mit einer medianen Überlebenszeit von etwa 60 Monaten leben KRPCa-Patienten mit Metastasen deutlich kürzer [9]. Internationale Leitlinien empfehlen die Fortführung der ADT während der KRPCa-Behandlung [2, 10, 14]. Eine Querschnittanalyse von Daten aus verschiedenen europäischen Ländern, darunter auch Deutschland, ergab jedoch, dass diese Leitlinien in der Routinepraxis offenbar nicht konsequent befolgt werden [11, 16].

Die primären Ziele dieser Studie waren die retrospektive Bestimmung des Anteils der PCa-Patienten, die die ADT nach Beginn der Kastrationsresistenz in der medizinischen Routineversorgung in Deutschland fortsetzen und die Beschreibung der Behandlungsmuster. Sekundäre Ziele waren Behandlungsdauer und Zeit bis zur Kastrationsresistenz [1, 4].

## Methodik

### Studiendesign und Datenquelle

Für diese retrospektive Beobachtungsstudie wurden Daten aus dem ehemaligen Online-Portal „uroscience.de“ verwendet, das vom Berufsverband der Deutschen Urologen (BvDU), dem Deutschen Institut für Versorgungsforschung (DIFA) und der Litixsoft GmbH Leipzig im Rahmen des Projekts UROgister/UROscience entwickelt worden war [1, 4]. Das Projekt UROgister gab allen deutschen Urologen eine einfach zu bedienende und sichere Möglichkeit, die verpflichtende Meldung an das regionale Krebsregister durchzuführen [1]. Die Datenbank UROscience bestand aus anonymisierten Daten aus den elektronischen Krankenakten, die im klinischen Alltag gesammelt wurden und bot Urolog*innen und anderen Beteiligten die Möglichkeit, an medizinischen Forschungsprojekten teilzunehmen [1, 4]. Für diese Analyse wurden am 10. Oktober 2020 Daten aus der Datenbank abgerufen. Diese umfassen 609.308 ambulante Patienten aus 32 urologischen Praxen, die zwischen dem 01.01.2011 und dem 31.03.2020 registriert wurden (s. Suppl. Abb. 1). Der Prozess der Patientenauswahl ist im Suppl. Tab. 1 dargestellt. Ein Flussdiagramm der Ein- und Ausschlüsse ist im Suppl. Abb. 2 dargestellt.

### Studienpopulation

In der Studie wurden die folgenden Ein- und Ausschlusskriterien angewandt: männliche Patienten im Alter von ≥ 18 Jahren bei Diagnosestellung; Diagnose eines PCa (ICD-10-CM C61 mit Zusatzkode „G“ [bestätigte Diagnose] oder „Z“ [Zustand nach der Diagnose]) zwischen dem 01.01.2011 und dem 31.12.2015; Beginn einer ADT-Therapie zwischen dem 01.07.2011 und dem 31.12.2015 nach einem Prä-Indexzeitraum von 6 Monaten ohne ADT und Medikation, die auf KRPCa hinweist (um Patienten auszuwählen, deren ADT ausschließlich während des Indexzeitraums begonnen wurde). Nach Anwendung dieser Kriterien wurden insgesamt 3.112 Patienten, die eine primäre ADT erhielten, in die Studie aufgenommen und als „PADT“-Gruppe bezeichnet. Die Gesamtzahl der Patienten, die nach einer primären ADT eine Sekundärtherapie erhielten, die auf eine Kastrationsresistenz hinwies, betrug 453; sie wurden als „KRPCa“-Gruppe bezeichnet.

### Behandlung und Endpunkte der Studie

Die ADT wurde über die Verschreibung von GnRH-Agonisten (Buserelin, Goserelin, Leuprorelin, Triptorelin), GnRH-Antagonisten (Abarelix [seit 2019 nicht mehr auf dem EU-Markt erhältlich], Degarelix), AA (Bicalutamid, Flutamid) oder einer Kombinationstherapie aus GnRH-Agonisten und AA, die zu einer maximalen Androgenblockade (MAB) führt, identifiziert. Zu den sekundären Behandlungen, die auf KRPCa hinweisen, gehörten die Verschreibung von Docetaxel, Cabazitaxel, Abirateron, Enzalutamid und Apalutamid.

Die Behandlungsmuster Unterbrechung, Wechsel, Abbruch und Fortführung der Behandlung waren für diese Studie von Interesse und wurden in weitgehender Übereinstimmung mit denen der retrospektiven Studie von Hupe et al. [5] verwendet, um einen Vergleich zu ermöglichen (Tab. 1).

**Tab. 1:** Behandlungsmuster bei PADT- (primäre Androgendeprivationstherapie) und KRPCa-Patienten (kastrationsresistentes Prostatakarzinom)

Behandlungsmuster	Definition
Unterbrechung der Behandlung	Keine ADT-/KRPCa-Verschreibung für einen Zeitraum von > 6 Monaten, aber eine weitere Verschreibung in derselben oder einer anderen Klasse während der verbleibenden Zeit der Nachbeobachtung
Wechsel der Behandlung	Die Verschreibung eines ADT-/KRPCa-Medikaments, das einen anderen Wirkstoff als das vorherige Medikament enthält, unabhängig von der Zeit, die zwischen den beiden Verschreibungen liegt. Ein Wechsel zurück zu einem früheren Medikament wurde nicht als Wechsel gewertet
Abbruch der Behandlung	Ein Zeitraum von > 6 Monaten ab dem Datum der letzten verfügbaren ADT-/KRPCa-Verschreibung bis zum Ende der Nachbeobachtung, ohne weitere Verschreibung
Fortführung der Behandlung	Ein Zeitraum von ≤ 6 Monaten ab dem Datum der letzten verfügbaren ADT-/KRPCa-Verschreibung bis zum Ende der Nachbeobachtung bzw. Wechsel auf eine andere Therapie (im Gegensatz zum Absetzen)

*ADT* Androgendeprivationstherapie, *KRPCa* kastrationsresistentes Prostatakarzinom

Die in dieser Studie berücksichtigten Endpunkte für die Zeit bis zum Ereignis waren TT („time on treatment“) für die ADT- und KRPCa-Behandlung und TTCR („time to castration resistance“). TT wurde definiert als die Zeit vom Tag der ersten Verschreibung bis zum Tag der letzten Verschreibung. Im Falle einer Unterbrechung oder eines Wechsels innerhalb desselben Therapietyps (andere ADT oder KRPCa-Medikamente) markierte die letzte Verschreibung nach der Unterbrechung oder dem neuen Wirkstoff das Ende der Behandlung. Die TTCR wurde vom Tag der ersten ADT-Verschreibung bis zum Tag der ersten Verschreibung des Medikaments, das eine Kastrationsresistenz anzeigt, gemessen.

### Statistische Analyse

Die deskriptiven Statistiken umfassten Mittelwert, Standardabweichung (SD), Median und Interquartilsbereiche (Q1, Q3) für quantitative Variablen sowie Anzahl und Prozentsätze für kategoriale Variablen. Die Ereigniszeitanalysen (TT für ADT und KRPCa und TTCR) wurden mit der Kaplan-Meier-Methode durchgeführt und der entsprechende Median mit 95-%-Konfidenzintervallen (KI) berichtet. Wurde dieser nicht erreicht, wurde der Median mit den Interquartilsbereichen angegeben. Alle Analysen wurden mit SAS® Version 9.4 (SAS Institute Inc., Cary, North Carolina, USA) durchgeführt.

## Ergebnisse

### Patientencharakteristika

**Abb. 1:**
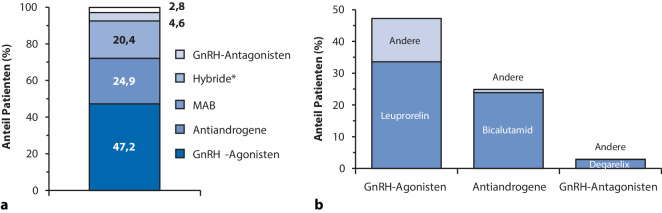
Die meistverschriebenen Medikamente, aufgeschlüsselt nach Androgendeprivationstherapie (ADT)-Klasse (**a**) und individuell für die einzelnen Wirkstoffe (**b**). *Sternchen:* Bei Hybriden bezieht sich die Zulassung auf ein Originalprodukt, wobei der Stoff jedoch nicht alle Kriterien für ein Generikum erfüllt und daher zusätzliche vorklinische und klinische Daten vorgelegt werden müssen. *MAB* maximale Androgenblockade, *GnRH* Gonadotropin-Releasing-Hormon

Die Baseline-Charakteristika der PADT-Patienten, stratifiziert nach ADT-Klassen, sind in Tab. 2 zusammengefasst. Das Durchschnittsalter in der gesamten PADT-Gruppe betrug bei Behandlungsbeginn 75,5 (±8,0) Jahre, und bei 24,6 % der Patienten war mindestens eine Komorbidität dokumentiert. Der Anteil der Patienten mit dokumentierten Komorbiditäten war bei Beginn der ADT (24,1 %) und bei Beginn des KRPCa (23,6 %) in der KRPCa-Gruppe ähnlich. Die meistverschriebenen Medikamente nach ADT-Klasse sind in Abb. 1a dargestellt, aufgeschlüsselt nach einzelnen Wirkstoffen in Abb. 1b.

**Tab. 2:** Patientencharakteristika in der PADT-Gruppe (primäre Androgendeprivationstherapie)

	Gesamt-PADT	GnRH-Agonisten	Hybride	GnRH-Antagonisten	Nichtsteroidale Antiandrogene	MAB
Patienten (*n* [%])	3112 (100)	1470 (47,2)	144 (4,6)	88 (2,8)	774 (24,9)	636 (20,4)
*Alter bei Beginn der ADT (Jahre)*^1^						
Mittelwert (SD)	75,5 (8,0)	76,4 (7,5)	76,2 (8,2)	73,3 (8,1)	74,7 (8,5)	74,7 (8,0)
Median (Q1, Q3)	76 (71,81)	77 (72, 82)	77 (72, 82)	74,5 (68, 79,5)	75 (70, 80)	75 (71, 80)
< 50 (%)	6 (0,2)	0 (0)	0 (0)	0 (0)	3 (0,4)	3 (0,5)
50–59 (%)	119 (3,8)	38 (2,6)	7 (4,9)	4 (4,5)	41 (5,3)	29 (4,6)
60–69 (%)	491 (15,8)	196 (13,3)	20 (13,9)	25 (28,4)	146 (18,9)	104 (16,4)
70–79 (%)	1516 (48,7)	711 (48,4)	68 (47,2)	37 (42,0)	366 (4,3)	334 (52,5)
80–89 (%)	894 (28,7)	485 (33,0)	45 (31,3)	21 (23,9)	195 (25,2)	148 (23,3)
≥ 90 (%)	86 (2,8)	40 (2,7)	4 (2,8)	1 (1,1)	23 (3)	18 (2,8)
*Krankenversicherung*						
Gesetzliche Krankenversicherung (*n* [%])	2754 (88,5)	1340 (91,2)	131 (91)	73 (83)	656 (84,8)	554 (87,1)
Private Krankenversicherung (*n* [%])	358 (11,5)	130 (8,8)	13 (9)	15 (17)	118 (15,2)	82 (12,9)
*Komorbiditäten bei Start der ADT*						
Ja (*n* [%])	765 (24,6)	395 (26,9)	27 (18,8)	20 (22,7)	184 (23,8)	139 (21,9)
Nein (*n* [%])	2347 (75,4)	1075 (73,1)	117 (81,3)	68 (77,3)	590 (76,2)	497 (78,1)
*Art der Komorbiditäten bei Start der ADT*^2‑3^						
Diabetes mellitus (*n* [%])	228 (7,3)	115 (7,8)	10 (6,9)	4 (4,5)	57 (7,4)	42 (6,6)
Hypertonie (*n* [%])	440 (14,1)	238 (16,2)	8 (5,6)	11 (12,5)	106 (13,7)	77 (12,1)
Adipositas (*n* [%])	65 (2,1)	42 (2,9)	1 (0,7)	2 (2,3)	14 (1,8)	6 (0,9)
Kardiovaskuläre Erkrankung (*n* [%])	309 (9,9)	178 (12,1)	9 (6,3)	5 (5,7)	68 (8,8)	49 (7,7)
Osteoporose (*n* [%])	18 (0,6)	6 (0,4)	0 (0)	1 (1,1)	8 (1)	3 (0,5)
Obstruktive Uropathie (*n* [%])	68 (2,2)	24 (1,6)	9 (6,3)	4 (4,5)	14 (1,8)	17 (2,7)

^1^Das Alter des Patienten zu diesem Zeitpunkt wurde anhand des Geburtsjahrs und des Jahres des Ereignisses von Interesse geschätzt

^2^Die Patienten können mehrere Komorbiditäten aufweisen. Die Gesamtzahl der Komorbiditäten summiert sich daher nicht zur Gesamtzahl der Patienten

^3^ICD-10-Codes: Diabetes (E10–14), Hypertonie (I10–15), Adipositas (E66), kardiovaskuläre Erkrankungen (I20–25, 142, 147–50, 163–66, 169.3, 169.4, 170, 173.9), Osteoporose (M80–82), obstruktive Uropathie (N13)

*GnRH* Gonadotropin-Releasing-Hormon, *PADT* primäre Androgendeprivationstherapie, *MAB* maximale Androgenblockade

### Fortführung der primären ADT nach Beginn der KRPCa-Therapie

Insgesamt 14,6 % der Patienten aus der PADT-Gruppe entwickelten ein KRPCa (*n* = 453), wobei 76,4 % dieser Patienten die primäre ADT fortsetzten, während sie eine sekundäre Behandlung begannen (*n* = 346). Der höchste Anteil an Patienten, die während der ADT ein KRPCa entwickelten, wurde bei denjenigen beobachtet, die GnRH-Antagonisten als primäre ADT erhielten (22,7 %).

### ADT-Behandlungsmuster

Insgesamt hatten die Patienten der PADT-Gruppe im Beobachtungszeitraum im Median 7 ADT-Verschreibungen (Q1, Q3: 3, 14). Im ersten Behandlungsjahr hatten Patienten in allen ADT-Klassen im Median 3 oder 4 Verschreibungen (Q1, Q3: 2, 4–5), mit Ausnahme der Patienten, die GnRH-Antagonisten erhielten (Median von 5 Verordnungen; Q1, Q3: 3, 7). Insgesamt hatten 13,4 % der Patienten in der PADT-Gruppe während des gesamten Beobachtungszeitraums nur eine ADT-Verschreibung. Während der gesamten Behandlungsdauer der Patienten waren GnRH-Agonisten die am häufigsten verschriebene ADT-Gruppe (91,0 %), gefolgt von AA (43,6 %), Behandlungen mit MAB (33,1 %) und GnRH-Antagonisten (5,0 %). Die mediane Anzahl der ADT-Verschreibungen im Beobachtungszeitraum war bei Patienten, die ein KRPCa entwickelten, doppelt so hoch wie bei denen, die kein KRPCa entwickelten (12 [Q1, Q3: 7, 19] vs. 6 [Q1, Q3: 2, 13]).

Wechsel, Unterbrechungen und Abbrüche der ADT innerhalb der PADT-Gruppe sind in Tab. 3 dargestellt. Beim Wechsel der primären ADT wurde der geringste Anteil bei den Patienten beobachtet, die mit GnRH-Agonisten behandelt wurden (36,7 %; im Vergleich dazu: Hybride 40,3 %; Antagonisten 53,4 %; AA 58,5 %; MAB 82,5 %). Vor dem Wechsel waren die Patienten, die GnRH-Agonisten erhielten, im Median 16,8 Monate auf ihrer ersten Therapie (Q1, Q3: 6,2, 34,1); Patienten mit GnRH-Antagonisten wechselten im Median nach 6,9 Monaten (Q1, Q3: 3,7, 17,5); Patienten mit AAs und MAB-Therapie wechselten im Median nach 2,2 Monaten (Q1, Q3: 0,5, 12,2) bzw. 3,1 Monaten (Q1, Q3: 1,7, 3,9). Die meisten Patienten der PADT-Gruppe, die ihre ursprüngliche ADT umstellten, wechselten zu GnRH-Agonisten (51,4 %) und AA (24,5 %), insbesondere zu Leuprorelin (32,0 %) und Bicalutamid (23,3 %). Die Unterbrechungsdauer lag im Median über alle ADT hinweg zwischen 7,4 (Q1, Q3: 6,3, 11,5) für die Patienten mit GnRH-Antagonisten und 8,5 Monaten (Q1, Q3: 6,4, 13,6) für die Patienten mit AAs als primäre ADT. Nach dem Absetzen der Behandlung erhielten die meisten Patienten (90,7 %) bis zum Ende des Nachbeobachtungszeitraums keine andere Therapie.

**Tab. 3:** Wechsel, Unterbrechungen und Abbrüche der ADT in der PADT-Gruppe (primäre Androgendeprivationstherapie) und der KRPCa-Behandlung (kastrationsresistentes Prostatakarzinom) in der KRPCa-Gruppe

	PADT-Gruppe (*n* = 3112)		KRPCa-Gruppe (*n* = 453)	
	Patienten (*n* [%])	Mediane Zeit bis zum ersten Ereignis, Monate (Q1, Q3)	Patienten (*n* [%])	Mediane Zeit bis zum ersten Ereignis, Monate (Q1, Q3)
*Wechsel*				
≥ 1 Wechsel	1623 (52,2)	4,1 (1,7, 18,4)	144 (31,8)	10,6 (5,2, 18,6)
≥ 2 Wechsel	757 (24,3)	–	7 (1,5)	–
*Unterbrechungen*				
≥ 1 Unterbrechung	1329 (42,7)	3,3 (0,0, 10,5)	60 (13,2)	5,5 (1,4, 17,2)
≥ 2 Unterbrechungen	750 (24,1)	–	12 (2,6)	–
*Abbrüche*				
Abbrüche	2558 (82,2)	17,8 (3,6, 39,2)	345 (76,2)	7,3 (1,8, 16,4)

*PADT* primäre Androgendeprivationstherapie, *KRPCa* kastrationsresistentes Prostatakarzinom

### KRPCa-Behandlungsmuster

Abirateron und Enzalutamid waren mit 67,3 % und 58,1 % der Patienten, die ein KRPCa entwickelten, die am häufigsten verschriebenen sekundären Hormonbehandlungen. Nur für 5,5 % der Patienten wurde eine nachfolgende Chemotherapie dokumentiert, insbesondere Docetaxel (5,3 %) und Apalutamid (2,0 %). Insgesamt erhielten die KRPCa-Patienten im Beobachtungszeitraum im Median 8 Verordnungen (Q1, Q3: 3, 16) einer KRPCa-Behandlung. Im Allgemeinen erhielten die meisten Patienten Abirateron als erste Sekundärbehandlung für KRPCa (58,1 %), gefolgt von Enzalutamid (36,2 %). Die Wechsel, Unterbrechungen und Abbrüche der KRPCa-Therapien innerhalb der KRPCa-Gruppe sind ebenfalls in Tab. 3 aufgeführt. Die Unterbrechungsdauer über alle KRPCa-Behandlungen hinweg lag im Median bei 9,2 Monaten (Q1, Q3: 8,0, 11,7).

### Dauer der Behandlung (TT) und Zeit bis zur Kastrationsresistenz (TTCR)

Insgesamt setzten 17,8 % der PADT-Gruppe die ADT innerhalb des Beobachtungszeitraums nicht ab. Die mediane TT mit der ADT betrug 25,9 (95 %-KI: 24,4–27,8) Monate, wobei sie bei den Patienten, die GnRH-Agonisten (TT: 27,6 [95 %-KI: 24,7–30,1] Monate) und AA (TT: 26,3 [95 %-KI: 24,0–31,0] Monate) als primäre ADT erhielten, am längsten und bei den Patienten mit GnRH-Antagonisten (TT: 21,2 [95 %-KI: 14,0–25,3] Monate) am kürzesten war. Die Wahrscheinlichkeit, die ADT fortzuführen, nahm im Laufe der Zeit stetig ab, von 40,7 % nach 3 Jahren auf 20,1 % nach 6 Jahren und 12,7 % nach 8 Jahren, mit den niedrigsten Werten in der Gruppe der GnRH-Antagonisten (26,1 %/14,6 %/5,5 % nach 3/6/8 Jahren). Insgesamt entwickelten 85,4 % der PADT-Gruppe während des Beobachtungszeitraums kein KRPCa. Die Berechnung der medianen Zeit zwischen ADT-Beginn und Beginn der KRPCa-Therapie ergab einen Wert von 29,9 Monaten (Q1, Q3: 15,1, 48,5). Die geschätzten Wahrscheinlichkeiten, 3, 6 und 8 Jahre nach Behandlungsbeginn ein KRPCa zu entwickeln, lagen bei 11,1 %, 20,1 % bzw. 25,9 %. Am höchsten war sie in der Gruppe der GnRH-Antagonisten mit 19,4 %, 36,0 % und 36,0 % 3, 6 und 8 Jahre nach Beginn der ADT. Die mediane TT der KRPCa-Behandlung betrug 11,0 (95 %-KI: 8,8–13,0) Monate.

## Diskussion

Für diese retrospektive Real-world-Studie mit einer Nachbeobachtungszeit von fast 9 Jahren wurden Daten des Webservers UROscience – basierend auf elektronischen Patientenakten von niedergelassenen deutschen Urologen – analysiert, um der Frage nachzugehen, ob die ADT bei Auftreten eines KRPCa als Basistherapie fortgeführt wird.

Etwa 15 % der Patienten in dieser Studie erhielten eine Sekundärbehandlung für KRPCa wie Chemotherapie oder Hormonbehandlung, was der geschätzten Prävalenz von 10–20 % der Prostatakrebspatienten entspricht, die innerhalb von 5 Jahren ein KRPCa entwickeln, wie an anderer Stelle berichtet wurde [7]. Im Falle der Chemotherapie ist zu erwähnen, dass insbesondere Docetaxel selten in urologischen Praxen, sondern eher in onkologischen Praxen verabreicht wird. Daher könnte die tatsächliche Zahl der Patienten, die Docetaxel als Zweitbehandlung erhalten, höher sein. Die mediane TTCR, definiert als die Zeit vom Beginn der ADT bis zur Sekundärbehandlung, betrug bei KRPCa-Patienten 29,9 Monate. In einer retrospektiven Analyse auf der Grundlage der Daten von PCa-Patienten, die eine ADT als Primärtherapie erhalten hatten [6], war die mediane Zeit bis zum Fortschreiten zum KRPCa ähnlich (38, Spanne: 4–158 Monate).

Nur etwa drei Viertel der Patienten, die eine KRPCa-Behandlung erhielten, führten ihre ADT fort. Diese Beobachtung deckt sich mit den Ergebnissen einer Studie, die Unterschiede in den KRPCa-Behandlungsmustern in fünf europäischen Ländern aufzeigt. Demnach erhielten 83 % der Patienten, bei denen in Deutschland ein KRPCa diagnostiziert wurde, weiterhin eine ADT [16]. In der Studie von Hupe at al. wurde bei 8–27 % der Patienten die ADT nach Beginn der Therapie mit einem neuen Wirkstoff abgesetzt [5]. Dabei empfehlen sowohl europäische als auch amerikanische Leitlinien die Fortführung der ADT nach Fortschreiten der Erkrankung zu einem metastasierten KRPCa [2, 10, 14] und auch in der Literatur besteht ein Konsens [3, 11]. Darüber hinaus wurde die Wirksamkeit aller kürzlich zugelassenen neuen Wirkstoffe für die Behandlung des progressiven PCa in klinischen Studien in Kombination mit ADT als „best supportive care“ gezeigt [3, 13].

Die Ergebnisse zur ADT stimmen mit denen der Beobachtungsstudie von Hupe et al. überein, die auf Daten von > 70 gesetzlichen Krankenkassen basierte [5]. Demnach war Leuprorelin in beiden Studien die am häufigsten verordnete primäre ADT. Auch der Anteil der Patienten, die die ADT nach der Erstverschreibung absetzten, war mit 13,4 % vergleichbar (11,2 % bei Hupe et al.). Die höhere Abbruchrate in unserer Studie im Vergleich zur Studie von Hupe et al. (82,2 % vs. 58,2 %) könnte auf den deutlich längeren Beobachtungszeitraum in unserer Studie (ca. 9 Jahre vs. 3 Jahre) zurückzuführen sein. Die Wahrscheinlichkeit, die ADT nach 3 Jahren abzusetzen, war in beiden Studien ähnlich (59,3 % vs. 58,2 % bei Hupe et al.).

Etwa drei Viertel der Patienten brachen die KRPCa-Therapie innerhalb des Beobachtungszeitraums ab, Grund hierfür könnte das Versterben der Patienten sein. Die Mehrheit der KRPCa-Patienten wurde mit Abirateron und Enzalutamid behandelt, nur ca. 5 % der Patienten erhielten eine Chemotherapie. Dies steht im Gegensatz zu der Studie von Hupe et al., in der fast der gleiche Anteil der Patienten mit Chemotherapie behandelt wurde wie mit Abirateron [5]. Wie oben beschrieben, könnte dies daran liegen, dass Docetaxel in urologischen Praxen seltener verabreicht wird und daher hier nicht erfasst wurde. Da nur Patienten aus der Auswertung ausgeschlossen wurden, die zeitgleich (am selben Tag) Docetaxel und ADT erhielten, wurde möglicherweise bei einigen Patienten Docetaxel, in Kombination mit ADT, auch zur Behandlung eines metastasierten hormonsensitiven PCa (mHSPCa) verabreicht.

Anonymisierte Datenbanken, die aus elektronischen Patientenakten abgeleitet werden, bieten einen großen Pool medizinischer Routinedaten, die für die Analyse mehrerer Indikationen verwendet werden können und daher eine äußerst wertvolle Quelle für Real-world-Daten darstellen. Allerdings sind bei Analysen basierend auf diesen Sekundärdaten auch naturgemäß Einschränkungen bei der Interpretation der Ergebnisse zu berücksichtigen. Bestimmte klinische Ereignisse wie Komorbiditäten wurden eventuell nicht vollständig dokumentiert. Darüber hinaus waren Daten, die für ein besseres Verständnis der Art und des Verlaufs der Krankheit relevant sein könnten, z. B. das Krankheitsstadium, Risikofaktoren wie Body Mass Index (BMI) oder Raucherstatus, Nebenwirkungen der Therapie und der Vitalstatus, nicht verfügbar. Informationen zu vorherigen oder begleitenden Therapien, etwa in Form von Arztbriefen, waren aufgrund der Anonymisierung nicht zugänglich, so dass z. B. Patienten mit operativen Eingriffen oder Strahlentherapie in der Erhebung nicht berücksichtigt werden konnten. Daher und auch aufgrund ihres deskriptiven Charakters müssen die Ergebnisse mit Vorsicht interpretiert werden, und es können keine Schlussfolgerungen zu Ursache-Wirkungs-Beziehungen gezogen werden.

## Fazit für die Praxis

- Die Fortführung der primären Androgendeprivationstherapie (ADT) beim Prostatakarzinom (PCa) nach Fortschreiten der Erkrankung zum kastrationsresistenten Prostatakarzinom (KRPCa) ist laut europäischen und amerikanischen Leitlinien empfohlen.
- Die hier präsentierte Studie basierend auf Daten von 3.112 PCa-Patienten aus urologischen Praxen mit einem Zeitraum von 9 Jahren hat gezeigt, dass in Deutschland die primäre ADT nur bei ca. 75 % der KRPCa-Patienten fortgeführt wird.
- Dieses Ergebnis zeigt, dass in Zukunft vermehrt auf eine Fortführung der primären ADT geachtet werden kann, um PCa-Patienten optimal und leitlinienkonform zu behandeln.

## Supplementary Information

Im Supplement wird das Studiendesign, mit den Zeitachsen, in Abb. 1 graphisch dargestellt.  In Tab. 1 werden die unterschiedlichen Studienpopulationen definiert und in Abb. 2 in einem  Flussdiagramm zusammengefasst, mit den jeweils ein- und ausgeschlossenen Patientengruppen.

## Data Availability

Die Studiendaten können auf begründete Anfrage zur Verfügung gestellt werden.
