# Supplementary material for: Androgen deprivation as initial and backbone therapy for prostate carcinoma cancer: A retrospective data analysis from urological practices in Germany
Source: Urologie. 2024 Aug 29;63(12):1251–8. [Article in German] doi: 10.1007/s00120-024-02434-z (PMC11618137; doi:10.1007/s00120-024-02434-z)
Supplement: Supplementary file 1 — Im Supplement wird das Studiendesign, mit den Zeitachsen, in Abb. 1 graphisch dargestellt. In Tab. 1 werden die unterschiedlichen Studienpopulationen definiert und in Abb. 2 in einem Flussdiagramm zusammengefasst, mit den jeweils ein- und ausgeschlossenen Patientengruppen. [file 120_2024_2434_MOESM1_ESM.docx]

**Supplement**

**Androgendeprivation als Initial- und Basis-Therapie beim Prostatakarzinom – Eine retrospektive Datenanalyse aus urologischen Praxen in Deutschland**

Peter J. Goebell^1^, Felix Cornelius^2^, Annika Fernandez Milano^3^, Sybill Hessler^4^, Matthias Schulze^5,*^

^1^Urologische und Kinderurologische Universitätsklinik, Friedrich-Alexander-Universität Erlangen-Nürnberg, Erlangen, Germany

^2^tumorscout, Germany

^3^IQVIA Commercial GmbH & Co. OHG, Frankfurt am Main, Germany

^4^Ipsen Germany, Munich, Germany

^5^Praxis Dr. Schulze, Markkleeberg, Germany

*Corresponding author: Dr. Matthias Schulze, [matthias.schulze@praxis-schulze.de](about:blank)

**Inhalte**

Suppl. Abbildungen 1 & 2

Suppl. Tabelle 1

**Suppl. Abbildung 1: Studiendesign**


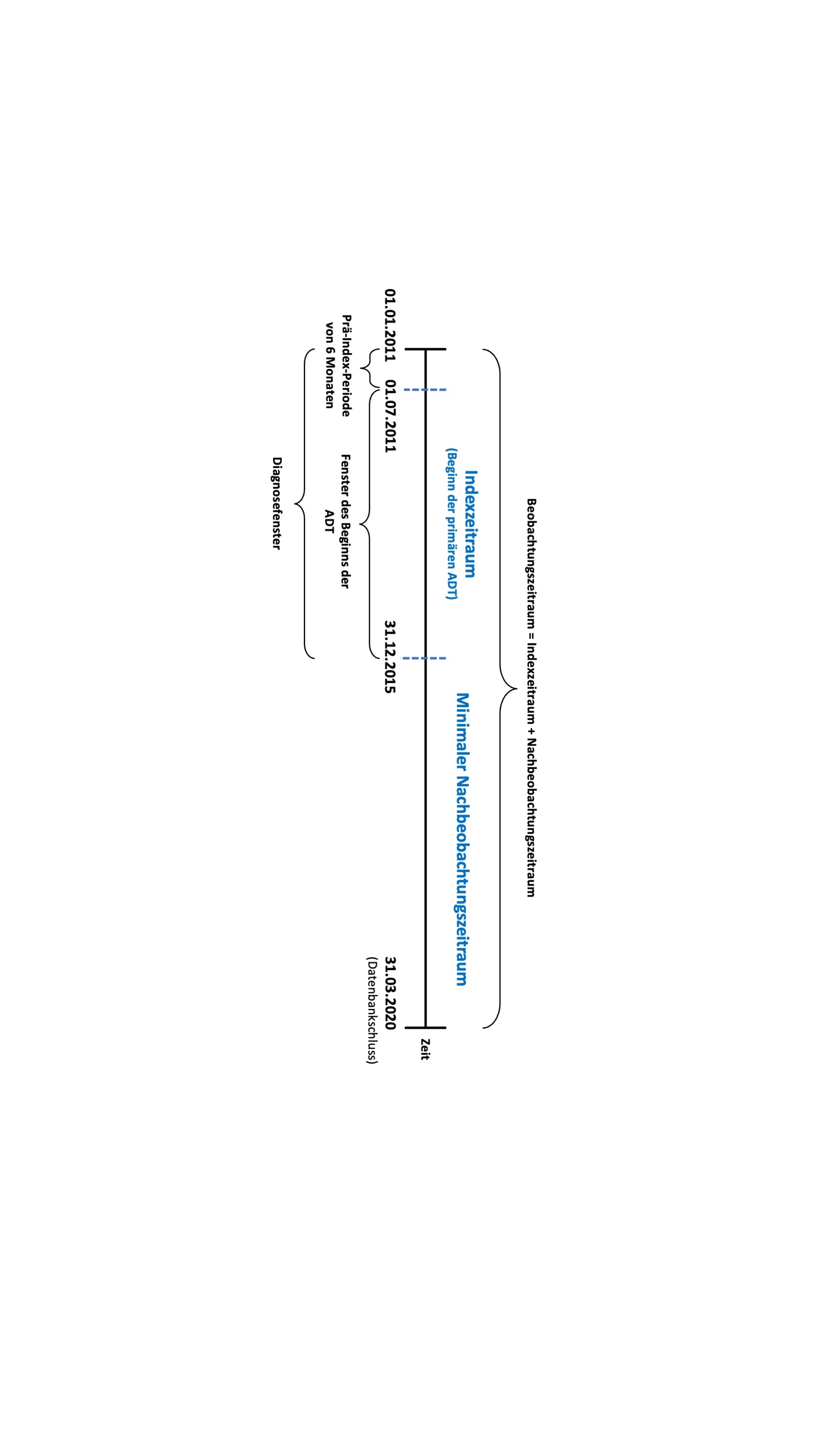


**Suppl. Tabelle 1: Studienpopulation**

| **Nr.** | **Definition der Population** | **Patienten verbleibend** | | **Patienten ausgeschlossen** | | |
| --- | --- | --- | --- | --- | --- | --- |
|  |  | **N** | **%*** | | **N** | **%*** |
| 1 | Alle Patienten in der Datenbank | 609.308 | - | | - | - |
| 2 | Männliche Patienten | 438.348 | 71,9 | | 170.960 | 28,1 |
| 3 | Patienten ≥18 Jahre mit Diagnose PCa zwischen 01.01.2011 und 31.12.2015 | 20.624 | 4,7 | | 417.724 | 95,3 |
| 4 | Patienten mit ADT zwischen 01.01.2011 und 31.03.2020 | 6.048 | 29,3 | | 14.576 | 70,7 |
| 5 | Patienten mit einer Prä-Index-Periode von 6 Monaten | 4.138 | 68,4 | | 1.910 | 31,6 |
| 6 | Patienten mit kontinuierlicher Erfassung | 3.814 | 92,2 | | 324 | 7,8 |
| 7 | Patienten mit ADT-Monotherapie oder Kombinations-therapie mit einer anderen ADT (Bicalutamid- oder Flutamid-Kombinationen) | 3.623 | 95,0 | | 191 | 5,0 |
| 8 | Patienten mit ADT begonnen vor 31.12.2015 | 3.112 | 85,9 | | 511 | 14,1 |
| 8.1 | Patienten mit kontinuierlicher ADT in den ersten 12 Monaten der Behandlung | 2.517 | 80,9 | | 595 | 19,1 |
| 8.2 | Patienten mit KRPCa nach Beginn der ADT | 420 | 16,7 | | 2.097 | 83,3 |
| 8.3 | Patienten mit laufender ADT bei KRPCa | 327 | 77,9 | | 93 | 22,1 |
| 9 | Patienten mit KRPCa nach Beginn der ADT | 453 | 14,6 | | 2.659 | 85,4 |
| 10 | Patienten mit laufender ADT bei KRPCa | 346 | 76,4 | | 107 | 23,6 |

*Der prozentuale Anteil der verbleibenden und ausgeschlossenen Patienten bezieht sich auf die vorherige Zeile als Referenzpopulation; ADT: Androgendeprivationstherapie; KRPCa: Kastrationsresistentes Prostatakarzinom; PCa: Prostatakarzinom

**Suppl. Abbildung 2: Flussdiagramm für Einschluss und Ausschluss von Patienten**

**
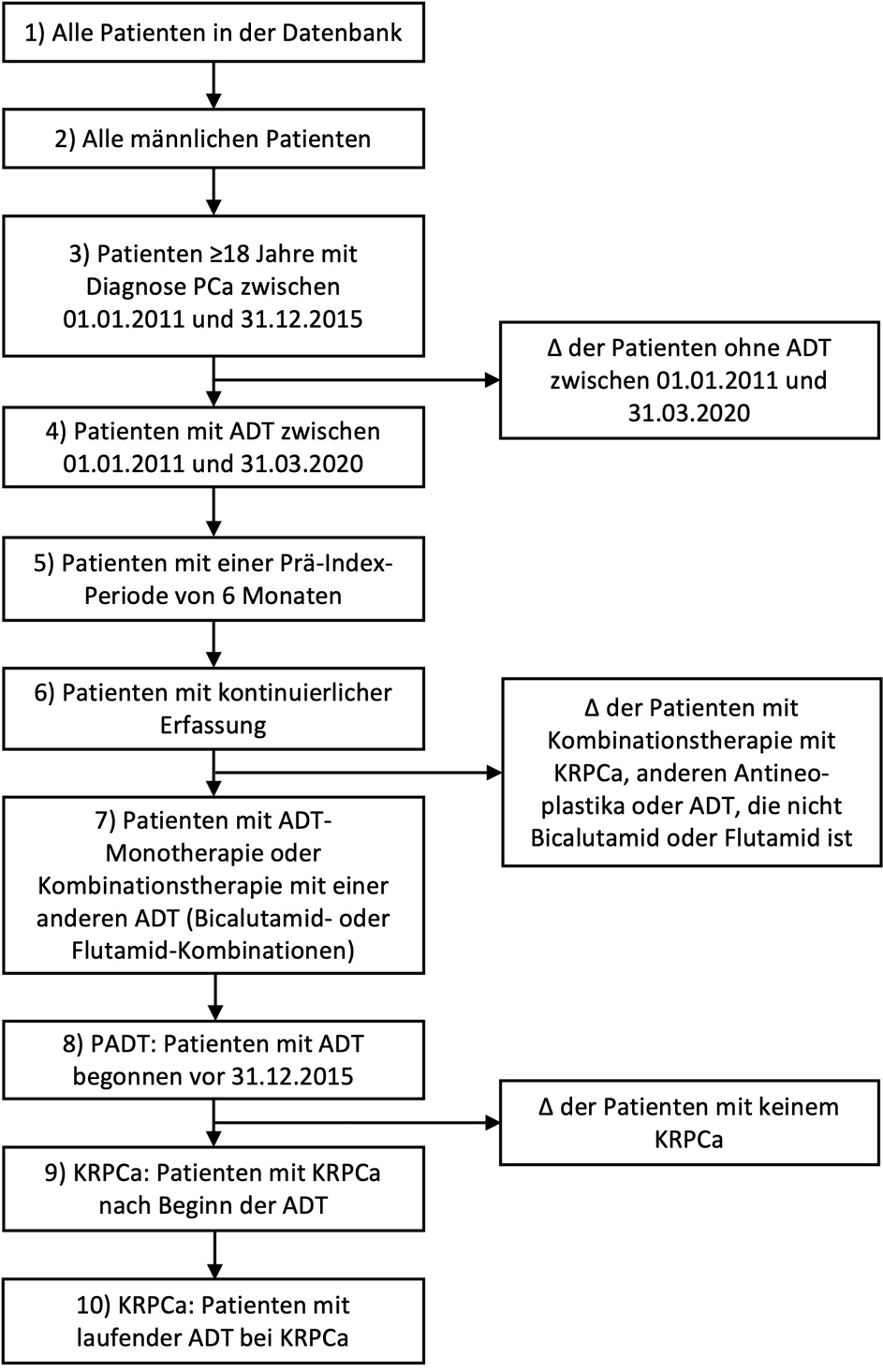
**
